# Chemical characterization and antioxidant properties of products and by‐products from *Olea europaea* L.

**DOI:** 10.1002/fsn3.1142

**Published:** 2019-08-10

**Authors:** Gabriella Tamasi, Maria Camilla Baratto, Claudia Bonechi, Anastasiya Byelyakova, Alessio Pardini, Alessandro Donati, Gemma Leone, Marco Consumi, Stefania Lamponi, Agnese Magnani, Claudio Rossi

**Affiliations:** ^1^ Department of Biotechnology, Chemistry and Pharmacy University of Siena Siena Italy; ^2^ Centre for Colloid and Surface Science (CSGI) University of Florence Firenze Italy; ^3^ National Interuniversity Consortium of Materials Science and Technology (INSTM) Firenze Italy; ^4^ Operative Unit University of Siena Calabria Italy

**Keywords:** Antioxidant, by‐products, *Olea europaea* L., pomace

## Abstract

The products and by‐products of *Olea europaea* L.: olive fruits (primary agricultural product), oils (primary agro‐industrial product), pomaces (agro‐industrial processing by‐product), and leaves (agricultural practices by‐product), are promising sources of bioactive compounds. In the present study, qualitative and quantitative analyses of selected bioactive components in olive fruits, oils, and pomaces were performed. Total polyphenol content and antioxidant activity were analyzed in all samples (humid pomaces 2015: TPP, 26.0 ± 1.5–43.7 ± 3.0 g(GAEq)/kg DW; TEAC/ABTS, 189.5 ± 3.7–388.1 ± 12.0 mmol(Trx)kg DW). Radical (DPPH) quenching potential was analyzed via photometric and EPR methods, obtaining Vis/EPR signal ratio by 1.05 ± 0.45 and 1.66 ± 0.39 for fruits and pomaces, respectively. Through HPLC‐UV and HPLC‐MS/MS techniques, oleuropein and hydroxytyrosol, as well as selected hydroxycinnamic acids and flavonoids, were identified and quantified in olive fruits and pomaces. The main components were rutin, luteolin, and chlorogenic acid. Cytotoxic assay on fibroblast cells revealed toxic effects for selected extracts at highest tested concentrations (5%).

## 1 INTRODUCTION

*Olea europaea* L. is one of the most diffuse plants in the Mediterranean area, from which the extra‐virgin olive oil (EVOO) is traditionally obtained by cold pressing of olive fruits. EVOO represents the most used dressing in the Mediterranean diet, and many studies report its beneficial effects on human health (Battino et al., 2018; Buckland & Gonzalez, 2015; Covas, Torre, & Fitó, 2015; Delgado‐Lista et al., 2016; Estruch et al., 2018; Finicelli et al., 2018; Guasch‐Ferré et al., 2014; Khalatbary, 2013; Konstantinidou et al., 2010; Pérez‐Martínez, García‐Ríos, Delgado‐Lista, Pérez‐Jiménez, & López‐Miranda, 2011). This last aspect has received increased interest by the consumers of functional foods (containing biologically active minor components; Bonechi et al., 2017; Bonechi et al., 2018). The quality standards for EVOO are currently based on the combined evaluation of raw agricultural product, varietal and geographic characterization, organoleptic properties and sensory consumer expectations, and health‐related characteristics, such as high concentration of oleic acid, and content of bioactive components (at low percentage). The oxidative stability of EVOO is directly related to the presence of minor antioxidant components, which are also responsible for its main organoleptic properties (such as the spice, bitter taste, distinguishing the freshly milled product), as well as its health‐related properties, as preventing agents for cardiovascular diseases, atherosclerosis, and heart attacks, and antitumoural activities against colon and breast cancer (Batarseh & Kaddoumi, 2018; Bulotta et al., 2014; Rigacci & Stefani, 2016). Recently, olive polyphenols have been recognized as “health claims” by the EFSA, EU (Source: www.efsa.europa.eu). To this extent, it is very important to underline that antioxidant species, like carotenoids, tocopherols, and vitamins, can be found in many different foods (i.e., vegetables, cereals; Tamasi et al., 2015; Tamasi et al., 2019; Van Hung, 2016), whereas specific hydrophilic (poly)phenolic compounds (i.e., iridoids and secoiridoids, such as tyrosol, hydroxytyrosol, oleuropein, and ligstroside) are present, in great amount, only in EVOO, and related by‐products of olive oil production.

The production of olive oil presents a challenge to agro‐industrial waste management. Humid pomace, dry pomace, and mill wastewaters are produced in different quantities, based on specific milling technology. Mediterranean countries produce about 30 × 10^6^ m^3^ of mill waste. These by‐products are pathogen‐free and very rich in organic matter, in nutrients, and are also characterized by high levels of bioactive molecules (particularly, polyphenols), showing strong antimicrobial and phytotoxic activities and not easily biodegradable. For those bioactive properties, these by‐products can be recovered and reused for the production of functional foods for human or animal consumption, as well as for diet supplements and cosmetics formulations (Gullón et al., 2018; Herrero et al., 2011; Kishikawa et al., 2015; Di Nunzio et al., 2018; Romero, Medina, Mateo, & Brenes, 2018; Sousa, Costa, Alexandre, & Prata, 2019; Vitali Čepo et al., 2018). For that reason, the use of the phenolic compounds extracted from olive by‐products represents a great opportunity for the circular economy. Particular attention has been recently devoted to optimize nonconventional extraction procedures able to produce high‐quality phytocomplexes by using nontoxic solvents. These protocols are usually assisted by ultrasound, microwave, or supercritical fluid extraction (SFE) using carbon dioxide as solvent (Chanioti & Tzia, 2018; Herrero, Pilar Sánchez‐Camargo, Cifuentes, & Ibáñez, 2015; Xie et al., 2019). Several studies also indicate the possibility to increase the stability and the bioavailability of antioxidant and natural bioactive molecules using new carrier systems, like liposomes or polymeric micelles’ formulations (Bonechi et al., 2018; Leone et al., 2018, 2016; Zhang, Huang, & Li, 2014).

Given this opportunity, the present study explored the chemical and nutraceutical characterization of products and by‐products of *O. europaea* L.: olive fruits (primary agricultural products), EVVOs (primary agro‐industrial products), pomaces (by‐products from agro‐industrial processing), collected at the harvestings in 2014–2015. Particular attention was devoted to the evaluation of radical scavenging activity, via Trolox‐equivalent antioxidant capacity assays (TEAC) following the quenching of two different radicals, ABTS cation, through UV‐Vis spectrophotometry, and neutral DPPH, through UV‐Vis and EPR techniques (Polovka, 2006; Prior, Wu, & Schaich, 2005; Thaipong, Boonprakob, Crosby, Cisneros‐Zevallos, & Byrne, 2006; Yu & Cheng, 2008). EPR spectroscopy is a straightforward tool for the radical determination, and it has been previously applied for the determination of antioxidant activity of crude extracts and galloyl quinic derivatives, as well as antioxidant defense and oxidative damage processes evaluation against stress conditions (Baratto et al., 2003; Fini et al., 2014; Gori et al., 2016; Megar et al., 2009). Selected polyphenols have been also identified and quantified through HPLC‐UV and HPLC‐MS/MS techniques, optimizing the analytical protocols on the basis of the chemical properties of the matrix and analytes.

The approach presented in this study focuses on the valorization of primary and secondary products from *O. europaea* L., highlighting the possibility to utilize the pomaces as source of bioactive molecules. This represents a challenge and a great opportunity from both environmental and economical points of view, building a model to increase the sustainability of agricultural and agro‐industrial productions.

## 2 MATERIALS AND METHODS

### 2.1 Reagents, standards, and solvents

All reagents and standards were analytical grade, and were used as purchased from Sigma‐Aldrich: Folin–Ciocalteu's phenol reagent, sodium carbonate, gallic acid, potassium persulfate, ABTS (2,2'‐azino‐bis(3‐ethylbenzthiazoline‐6‐sulphonic acid)), DPPH (2,2‐diphenyl‐1‐picrylhydrazyl), Trolox (6‐hydroxy‐2,5,7,8‐tetramethylchroman‐2‐carboxylic acid), tyrosol, hydroxytyrosol, oleuropein, caffeic acid, ferulic acid, *p*‐Coumaric acid, chlorogenic acid, luteolin, luteolin‐7‐*O*‐rutinoside, quercetin dihydrate, rutin trihydrate, naringenin, genistein, and resveratrol. All solvents were gradient HPLC grade: methanol, ethanol, acetonitrile, formic acid, acetic acid, diethyl oxide, cyclohexane, and *n*‐hexane. Bi‐distilled water was produced by Acquinity P/7 distiller (MembraPure GmbH).

### 2.2 Sample collection and storage

All samples were collected at harvesting/milling time in 2014 and 2015, from oil milling plants in southwest Tuscany (names are not reported for privacy reasons; Table S1). The samples of olive fruits, olive oil (extra‐virgin), and pomace, coded as F1x‐Y, EVOO1x‐Y, and P1x‐Y, were related to the same farm/oil mill. All the oil milling plants were based on two‐phase technology (olive oil and humid pomace), except for samples 15‐A and 15‐C that were from three‐phase systems (olive oil, dry pomace, and vegetation water). In addition, the groups of samples (14‐A, 15‐D, 15‐E) and (15‐F, 15‐G, 15‐H, 15‐I, 15‐J) were collected from two distinct oil milling plants. EVOO samples were stored in the dark at −20 ± 1°C, until pretreatment or direct analyses. Olive fruits and pomace samples were freeze‐dried within 24 hr after collection (5Pascal Lio‐5P; usual working condition: condenser, −51 ± 2°C; pump pressure 1.2 ± 0.5 mbar; 72 hr) and stored (darkness, −20 ± 1°C) until subsequent pretreatment.

### 2.3 Sample pretreatments: extraction of antioxidant components

#### 2.3.1 Extra‐virgin olive oils (EVOOs)

Aliquots of 2.50 g of sample (analytically weighed, Radwag AC220/C/2, max capacity 100 g, readability 0.0001 g; Radom, Poland) were diluted by 12.5 ml of *n*‐hexane and then extracted by 5 ml of hydroalcoholic mixture (EtOH/H_2_O, 80/20%, v/v). The extraction was ultrasound assisted (10 min, 21 ± 2°C; power, 120 Watt; sound frequency, 35 kHz; ultrasonic bath Sonorex Bandelin), and the suspension was centrifuged (5 min, 1,882 g; Centrifuge Thermo Electron Corporation PK 110). The procedure was repeated two additional times, using 5 ml hydroalcoholic medium each (total volume extract, 15 ml). The extract was used as such.

#### 2.3.2 Olive fruits and pomaces

Aliquots of 0.250 g of each sample (analytically weighed) were defatted by 7 ml of *n*‐hexane (twice), and the liquid phase was discarded. The residual solid phase was then extracted (ultrasound assisted) by an hydroalcoholic mixture (EtOH/H_2_O, 80/20%, v/v; first extraction 5 ml, second and third extractions 2.5 ml; total volume extract, 10 ml). The extract was used as such, or dried under nitrogen flow (overnight) and then lyophilized. The dry extract was stored (darkness, −20 ± 1°C). Before HPLC analyses, the dry extract was reconstituted in 2 ml of solvent. In case of pomace samples, preliminary analyses comparing extracts with and without defatting process were carried out, obtaining results within 3% difference. Following this analysis, pomaces were usually extracted without previous defatting process.

### 2.4 Antioxidant activity assays

#### 2.4.1 Acidity analysis for EVOO samples

Total acidity for EVOOs was determined following the 1991 2568/1991 and 1830/2015 procedure. An aliquot by 3.0 g of oil was diluted by Et_2_O/EtOH mixture (75/25%, v/v) and was titrated by KOH 0.010 M ethanolic solution (previously standardized by standard HCl 0.010 M), using phenolphthalein as indicator. The results were expressed as oleic acid equivalent percentage (%OAEq).

#### 2.4.2 Folin–Ciocalteu assay: total polyphenol content (TPP)

Total polyphenols (TPP) were determined by spectrophotometric Folin–Ciocalteu method (Singleton, Orthofer, & Lamuela‐Raventós, 1999), with some modifications (Tamasi et al., 2019). UV‐Vis spectrophotometer used was a dual‐beam Perkin Elmer Lambda EZ 201, equipped with software PESSW 1.2 (Perkin Elmer). The instrumental spectra range was 190–1100 nm, and PMMA/UV grade cuvettes (Kartell; 10 mm optical pathway) were used. Briefly, 3.5 ml of filtered and diluted (if necessary) hydroalcoholic extracts was added to 0.1 ml of Folin–Ciocalteu reagent and 0.4 ml of Na_2_CO_3_ water solution (35%, w/v). The mixture was incubated for 30 min in the dark at 21 ± 2°C. Finally, the absorbance at 750 nm (Abs_750_) was recorded, against water. The calibration curves were recorded by using standard solutions of gallic acid in the linear range, 0.25–10.00 mg/L, (*R* ^2^ > 0.990 were accepted for analyses; Figure S1a). The results were expressed as mg gallic acid equivalent per kg of dried sample (mg(GAEq)/kg DW).

#### 2.4.3 Trolox‐Equivalent antioxidant capacity (TEAC) assays

Antioxidant activity was assayed by following the radical scavenger activity of free radicals ABTS^•+^ and DPPH^•^ according to procedures previously reported (Brand‐Williams, Cuvelier, & Berset, 1995; Re et al., 1999) with some modifications (Tamasi et al., 2019). The calibration curves were recorded by using standard solutions of Trolox, in the linear range, 0.20–20.00 µM (from a 0.55 mM mother solution in EtOH; *R* ^2^ > 0.990 were accepted for analyses; Figure S1b,c). The quenching percentage was calculated as (ABTS^•+^, ΔAbs_734_%; DPPH^•^, ΔAbs_517_%):

$$\Delta {\text{Abs}}_{734/517}\%=\left\{\left[1-\left({\text{Abs}}_{\text{Trolox/Sample}}/{\text{Abs}}_{\text{Blank}}\right)\right]\times 100\right\}$$

where Abs_Trolox/Sample_ is the absorbance of the radical solution treated with standards or samples, and Abs_Blank_ is the absorbance of the radical solution as such (ABTS^•+^or DPPH^•^ not treated solutions).

The results of TEAC were expressed as mmol Trolox equivalent per kg of dried sample (mmol(TrxEq)/kg DW).

#### 2.4.4 TEAC/ABTS assay

The ABTS^•+^ radical cation was prepared by incubation of a solution of ABTS (7 mM in EtOH) with a K_2_S_2_O_8_ solution (140 mM in water) overnight (darkness, 4 ± 1°C) and dilution in EtOH before use. A known volume of this solution was treated with Trolox standard solutions or a known amount of extract (diluted, if necessary). After 30 min of incubation in the dark, at 21 ± 2°C, the adsorption at 734 nm was recorded, against EtOH.

#### 2.4.5 TEAC/DPPH assays

A stock solution of DPPH^•^ (0.10 mM in MeOH) was freshly prepared and used within 4 hr. A known volume of DPPH^•^ solution was treated with Trolox standard solutions or a known amount of extract (diluted, if necessary). After 15 min of incubation in the dark, at 21 ± 2°C, the adsorption at 517 nm was recorded, against MeOH. In case of olive fruits and pomaces, the same experiment was carried out reading the DPPH^•^ solution (blank and treated) via EPR spectroscopy. EPR spectra were acquired on continuous‐wave X‐band (CW, 9GHz) using a Bruker E500 ELEXSYS Series spectrometer (Bruker, Italy), with the ER 4,122 SHQE cavity. EPR measurements were performed at 21 ± 1°C, 9.8 GHz microwave frequency, 0.1 mT modulation amplitude, and 4 mW microwave power. The sample was placed into a 3.0 mm ID × 4.0 mm OD, suprasil tube. In this case, stock solution of DPPH^•^ was prepared (1.0 mM in MeOH) and the final concentration of radicals in each sample was 0.45 mM. The acquisition of EPR signal was carried out 15 min after the addition of the antioxidant (Trolox or extract; darkness, 21 ± 2°C; Figure S1d), and the antioxidant activity was calculated by the relative decrease in area (instead of absorbance). The area of the EPR spectra was calculated by the double integral of the DPPH signal.

### 2.5 Chromatography analyses

Liquid chromatography was conducted for the identification and quantification of tyrosol, hydroxytyrosol, and oleuropein, by using the isocratic HPLC Varian ProStar 210 machine equipped with UV 9050 detector, managed by Varian Workstation software (Varian, Inc). For other hydroxycinnamic acids and flavonoid analysis, the HPLC‐MS method was optimized by using a HPLC Agilent 1,200 Series (Agilent Technologies) coupled with a mass spectrometer TSQ Quantum Access (Thermo Scientific), equipped with electrospray ion source (ESI) and triple quadrupole analyzer. The Xcalibur software (Thermo Scientific) managed the instrument and collected the data.

#### 2.5.1 HPLC‐UV: identification and quantification of tyrosol, hydroxytyrosol, and oleuropein

The chromatographic separation was carried out following Tamasi et al. (2015) with some modifications on the basis of the matrix/analytes. A reverse‐phase column (Phenomenex Luna C18, 5U, 250 × 4.6 mm, 5 μm particles, 100 Å pores) with safeguard precolumn (Phenomenex C18, 4 × 3.0 mm) was used, and the elution was isocratic with H_2_O (CH_3_COOH, 0.2%)/CH_3_CN (70:30, v/v) eluent, at 0.5 ml/min flow rate (21 ± 2°C). The injection volume was 20 µl, and the UV detector was set at 280 nm. The analytical determination was carried out via external calibration method, using resveratrol as internal standard and standard solutions of hydroxytyrosol and oleuropein in MeOH; retention times: *R*_t_, 6.46 min (hydroxytyrosol) and 10.60 min (oleuropein) (Figure S2). The linear calibration ranges (*R* ^2^ > 0.990) were 15–230 (hydroxytyrosol) and 30–900 mg/L (oleuropein). The tyrosol (*R*_t_, 7.5 min) was also identified, through standard additions, but not quantified, as it was present in trace concentrations. The limit of quantification (LOQ) and limit of detection (LOD) were 10 and 3 mg/L for hydroxytyrosol, and 15 and 5 mg/L for oleuropein.

#### 2.5.2 HPLC‐MS: identification and quantification of hydroxycinnamic acids and flavonoids

A reverse‐phase column (Phenomenex Kinetex biphenyl, 10 × 2.1 mm, 5 μm particles, 100 Å pore, shell‐core), with safeguard precolumn (Phenomenex Phenyl, 4 × 2.0 mm), thermostated at 35 ± 1°C, was used (Tamasi et al., 2019). The eluents were as follows: (A) H_2_O/HCOOH 0.1% (v/v) and (B) MeOH/HCOOH 0.1% (v/v), and the separation was performed through a linear gradient: 0–1.0 min, 10% B (isocratic); 1.0–11.0 min, from 10% to 90% B (linear); 11.0–14.5 min, 90% B (isocratic); 14.5–15.0 min, from 90% to 10% B (linear); 15.0–18.0 min, 90% B (isocratic). The elution flow rate was 0.5 ml/min, and the injection volume was 5 µl. The peaks were analyzed by ESI‐MS detector having a triple quadrupole analyzer. The ESI conditions were optimized for negative ion current mode, based on a standard solution of quercetin: nebulizer gas (N_2_) inlet pressure, 30 psi; temperature, 270°C; capillary voltage, 4,000 V; collision energy, 30 V. Full scan (Total Ion Current, TIC mode) data were acquired by scanning m/z, 150–1,000, to identify the analytes. The quantification of selected species was carried out via SIM (single ion monitoring) and SRM (selected reaction monitoring) methods. Selected MS/MS method parameters are reported in Table 1 and Figure S3. The analytical determination was carried out via external calibration, by using genistein as internal standard. Calibration showing correlation factors *R* ^2^ > 0.990 was accepted for analyses. The values for LOQ and LOD were also defined (Table 1).

**Table 1:** Selected HPLC‐MS/MS and calibration method parameters for hydroxycinnamic acids and flavonoid identification and quantification

	*R*_t_ (min)	PM	[M‐H]^‐^ (m/z)	MS^2^ (m/z)	Mode	Calibration range (mg/ml)	LOQ/LOD (mg/ml)
Hydroxycinnamic acids							
Caffeic acid	4.60	180.2	179	135	SRM	0.01–9.00	0.010//0.003
Chlorogenic acid	4.73	354.3	353	179; 191	MRM	0.05–10.00	0.020//0.007
*p*‐Coumaric acid	6.07	164.2	163	119	SRM	0.10–5.00	0.030//0.010
Ferulic acid	6.90	194.2	193	134	SRM	0.10–5.00	0.030//0.010
Flavonoids							
Rutin	7.16	610.5	609	301	SRM	0.02–9.00	0.010//0.003
Quercetin	8.55	302.2	301	151	SRM	0.02–2.00	0.010//0.003
Luteolin	8.96	286.2	285	–	SIM	0.02–9.00	0.010//0.003
Naringenin	9.50	272.2	271	151	SRM	0.02–9.00	0.010//0.004
Genistein (IS)	9.43	270.2	269	–	SIM		

### 2.6 Cytotoxicity assay

Hydroalcoholic (EtOH/H_2_O, 80/20%, v/v) extracts of EVOOs, olive fruits, and pomaces 2014 and 2015 were tested in vitro for cytotoxicity, on mouse fibroblast NIH3T3 cells, following the ISO 10995‐5:2009 protocol. Standard solutions of pure rutin, quercetin, and pure EtOH were also tested, for comparison reasons. Details on cell culture and cell viability procedures were those reported in Bonechi et al. (2018). Briefly, the fibroblasts NIH3T3 were propagated in Dulbecco's modified Eagle's medium (DMEM), supplemented with 10% fetal calf serum (FCS), 1% L‐glutamine–penicillin–streptomycin, and 1% MEM nonessential amino acid, and maintained at 37°C, in a humidified atmosphere (5% CO_2_). When at confluence, cells were washed with phosphate‐buffered saline solution (0.1 M, PBS) and separated using a trypsin–EDTA solution and centrifuged (118 g, 5 min). The pellet was resuspended and diluted in medium solution and added by different concentrations (0.5, 1.0, and 5.0%, v/v) of tested solutions (standards or extracts). After 24 hr of incubation, cell viability was evaluated by neutral red uptake. The incubation medium was removed, and cells were washed with prewarmed PBS; then, the neutral red medium (NR, 0.33 g in 100 ml sterile water, then diluted 100 times) was added and the samples were further incubated at 37°C, 95% humidity, 5.0% CO_2_ for 3 hr. After incubation, the cells were rinsed with prewarmed PBS, NR desorbing solution (glacial acetic acid:ethanol:H_2_O, 1:50:49%, v/v) was added and samples were shaked (20–45 min). Finally, the absorbance of each solution was recorded at 540 nm, within 5 min.

### 2.7 Statistical data treatment

Three samples were collected for each type, pretreatments (extracts) were performed in triplicate for each sample, and triplicate analyses were performed for all measurements in each extract. The results were reported as mean values and estimated standard deviations (esd, *n* = 27). Calculation was made by using Microsoft Office Excel 2007, implemented with regression analysis subroutine, and Origin Pro8 SR2, (v.0891, B891, OriginLab Corporation). The analysis of variance and Tukey's test were carried out to determine significant differences, and data showing *p*‐values < 0.05 were considered statistically significant. The linear regression studies were based on the Pearson correlation matrix, and *p*‐value was calculated at 95% confidence interval. A multivariate statistical analysis was also performed by using the Unscrambler X version 10.4 (Camo software), and principal component analysis (PCA) was computed on auto‐scaled variables for olive fruits and pomace samples.

## 3 RESULTS AND DISCUSSION

### 3.1 Antioxidant activity characterization of EVOOs, olive fruits, and pomaces

#### 3.1.1 EVOO samples

The total acidity values for EVOOs‐14 (Table 2) were relatively high. In two cases, they were higher than the maximum value for “extra‐virgin” (0.8% OAEq) defined by 1991 (2568/1991; 1830/2015). The 2014 harvest was influenced by infestation of *Bactrocera oleae* (olive fly). Better results were obtained for EVOOs‐15, for which the total acidity averaged 0.314 ± 0.070% OAEq.

**Table 2:** Values of total acidity %(OAEq), TPP mg(GAEq)/kg FW, and TEAC/ABTS mmol(TrxEq)/kg FW, for EVOO hydroalcoholic (EtOH/H2O, 80/20%, v/v) extracts

	Acidity	TPP	TEAC/ABTS
2014			
EVOO14‐A	0.29 ± 0.01^a^	517 ± 35^a^	1.61 ± 0.04^a^
EVOO14‐B	1.10 ± 0.01^b^	252 ± 15^b^	0.93 ± 0.05^b^
EVOO14‐C	1.01 ± 0.01^b^	268 ± 9^b^	0.89 ± 0.05^b^
2015			
EVOO15‐A	0.300 ± 0.005^a^	452 ± 7^c^	1.43 ± 0.04^a^
EVOO15‐B	0.385 ± 0.007^c^	516 ± 10^a^	2.10 ± 0.10^c^
EVOO15‐C	0.374 ± 0.004^c^	427 ± 8^d^	1.32 ± 0.09^a^
EVOO15‐D	0.383 ± 0.005^c^	576 ± 6^e^	2.37 ± 0.15^d^
EVOO15‐E	0.380 ± 0.009^c^	600 ± 15^f^	2.50 ± 0.11^d^
EVOO15‐F	0.239 ± 0.005^d^	514 ± 5^a^	1.90 ± 0.05^c^
EVOO15‐G	0.213 ± 0.007^e^	558 ± 9^e^	2.24 ± 0.10^d^
EVOO15‐H	0.217 ± 0.003^e^	489 ± 11^g^	1.78 ± 0.07^a^
EVOO15‐I	0.340 ± 0.003^f^	496 ± 8^g^	1.85 ± 0.05 ^a^
EVOO15‐J	0.310 ± 0.004 ^a^	503 ± 4 ^a^	2.04 ± 0.08 ^c^

Note

The values are reported as average ± esd, and different letters in the same column indicate significant differences (*p* < .05, Tukey's test).

**Figure 1:**
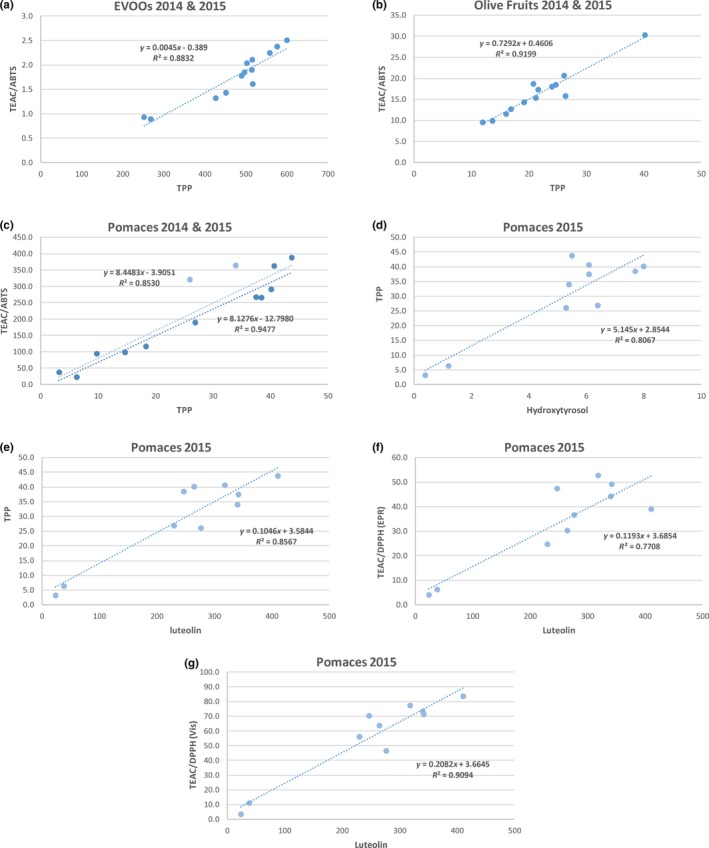
Linear correlation between antioxidant parameters and selected antioxidant components (hydroxytyrosol and luteolin). Analysis of significance showed *p* < .001 (95% confidence interval) for all data sets

Parameters of polyphenol content (TPP) and antioxidant capacity (TEAC/ABTS) followed similar year difference. The TPP parameters ranged from 452 ± 7 to 600 ± 15 mg(GAEq)/kg FW, with exception for EVOO14‐B/‐C, that showed lower values by 252 ± 15–268 ± 9 mg(GAEq)/kg FW (Table 2). A similar trend was revealed for TEAC/ABTS parameters that ranged 1.32 ± 0.09–2.50 ± 0.11 mmol(TrxEq)/kg FW for EVOOs‐15 and well compared with other studies (range 1.5–2.7 mmol(TrxEq)/kg FW; Pellegrini et al., 2003; Samaniego Sánchez et al., 2007). It is interestingly to note that reported vales were relevant to oils that were diluted without any preliminary extraction; thus, the TEAC values include the fat‐soluble antioxidant components. The TPP values were also well compared with data previously published (247–537 mg(GAEq)/kg, Šarolić et al., 2014; Galvano et al., 2007). A linear correlation between TPP and TEAC/ABTS parameters (Figure 1a) was found (y = 0.0045x‐0.3890; *R* ^2^ = 0.883; *p* < .001, at 95% confidence interval).

#### 3.1.2 Olive fruit samples

TPP values for olive fruits ranged 12.0 ± 0.9–40.2 ± 1.2 g(GAEq)/kg DW (Table 3), in agreement with previously reported studies of Tuscan cultivars (Frantoio, Rossellino, Ciliegino, Cuoricino, Grossolana; Romani, Mulinacci, Pinelli, Vincieri, & Cimato, 1999) and the Turkish Sarıulak variety (Arslan & Ozcan, 2011). Many factors can contribute to polyphenol content in fruits including variety, harvesting time, soil, and climatic conditions. As already mentioned, 2014 was an unusual year, with a *Bactrocera oleae* infestation in central Italy, and unusual weather conditions. The lower summer temperature and the higher summer humidity maximum *ca.* 22°C and 80%, respectively, (Figure S4) strongly influenced the fruit ripening process, producing a marked decrease in final quantity. On the other hand, warmer winter temperatures allowed higher quantity of insects to survive and lay eggs inside the fruits (Rice, 2000). On the contrary, the 2015 summer was hot and dry, and followed by a colder and dryer winter, leading to an increase in production and fruits’ quality.

**Table 3:** Values of TPP g(GAEq)/kg DW, TEAC/ABTS, and TEAC/DPPH mmol(Trx)/kg DW (for both photometric and EPR measurements) for olive fruit and pomace hydroalcoholic (EtOH/H2O, 80/20%, v/v) extracts

	TPP	TEAC/ABTS	TEAC/DPPH(Vis)	TEAC/DPPH(EPR)
Olive fruits				
F14‐A	19.2 ± 0.7^a^	14.3 ± 0.5^a^	55.5 ± 1.9^a^	62.5 ± 2.3^a^
F14‐B	12.0 ± 0.9^b^	9.5 ± 0.8^b^	16.0 ± 0.7^b^	14.2 ± 0.9^b^
F14‐C	13.7 ± 0.5^b^	9.9 ± 0.6^b^	14.7 ± 0.5^b^	37.1 ± 1.5^c^
F15‐A	16.9 ± 0.7^c^	12.6 ± 0.5^c^	50.2 ± 2.1^a^	44.5 ± 1.1^d^
F15‐B	26.1 ± 0.7^d^	20.6 ± 0.6^d^	78.4 ± 4.1^c^	57.2 ± 1.1^e^
F15‐C	21.2 ± 0.4^a^	15.3 ± 0.7^a^	62.7 ± 0.4^d^	45.5 ± 2.6^d^
F15‐D	16.0 ± 0.3^c^	11.5 ± 0.2^c^	40.4 ± 1.7^e^	23.0 ± 1.8^f^
F15‐E	24.7 ± 0.9^d^	18.4 ± 0.5^e^	55.2 ± 0.3^a^	36.5 ± 0.9^c^
F15‐F	40.2 ± 1.2^e^	30.3 ± 1.1^f^	60.6 ± 1.1^d^	121.1 ± 1.2^g^
F15‐G	24.0 ± 0.8 ^d^	18.0 ± 0.8 ^e^	75.8 ± 2.9 ^c^	53.5 ± 1.1 ^e^
F15‐H	21.6 ± 0.6^a^	17.3 ± 0.5^e^	67.3 ± 6.0^f^	85.3 ± 9.8^hr^
F15‐I	20.8 ± 0.2^a^	18.7 ± 0.1^e^	74.7 ± 0.6^c^	69.4 ± 0.8^hr^
F15‐J	26.4 ± 0.5^d^	15.8 ± 0.3^a^	38.0 ± 0.1^e^	117.6 ± 6.2^g^
Pomaces				
P14‐A	18.3 ± 0.6^a^	116.2 ± 8.6^a^	63.3 ± 2.0^a^	42.2 ± 1.7^a^
P14‐B	14.7 ± 1.0^a^	98.0 ± 3.1^b^	71.3 ± 1.7^b^	37.5 ± 2.4^a^
P14‐C	9.8 ± 0.6^b^	93.6 ± 1.3^b^	69.2 ± 2.1^b^	40.7 ± 2.1^a^
P15‐A	6.3 ± 0.5^c^	23.0 ± 2.8^c^	11.0 ± 0.2^c^	6.2 ± 0.2^b^
P15‐B	26.9 ± 1.2^d^	189.5 ± 3.7^d^	55.9 ± 1.6^d^	24.7 ± 0.3^c^
P15‐C	3.2 ± 0.5^e^	37.2 ± 2.7^e^	3.2 ± 0.1^e^	3.9 ± 0.5^d^
P15‐D	38.4 ± 3.3^f^	265.4 ± 10.1^f^	70.3 ± 0.9^f^	47.3 ± 1.6^e^
P15‐E	40.1 ± 2.2^f^	290.5 ± 9.2^g^	63.6 ± 3.0^g^	30.3 ± 3.2^f^
P15‐F	37.5 ± 1.9^f^	267.4 ± 19.3^f^	71.3 ± 5.5^f^	49.1 ± 0.7^e^
P15‐G	40.6 ± 0.5^f^	362.0 ± 18.2^hr^	77.1 ± 3.8^hr^	52.8 ± 3.4^e^
P15‐H	43.7 ± 3.0^g^	388.1 ± 12.0^hr^	83.4 ± 7.3^hr^	39.0 ± 1.0^g^
P15‐I	33.9 ± 0.1^hr^	364.5 ± 9.5^hr^	73.3 ± 1.0^f^	44.2 ± 4.9^hr^
P15‐J	26.0 ± 1.5^d^	321.2 ± 15.4^g^	46.4 ± 0.1^i^	36.6 ± 0.3^g^

Note

The values are reported as average ± esd (*n* = 27), and different letters in the same column (among the same matrix) indicate significant differences (*p* < .05, Tukey's test).

A similar trend was found in the TEAC/ABTS and TEAC/DPPH data. TEAC/ABTS values ranged 9.5 ± 0.8–30.3 ± 1.1 mmol(TrxEq)/kg DW, resulting from two to six times higher with respect to values reported for a Turkish variety (4.7 mmol(TrxEq)/kg DW; Arslan & Ozcan, 2011; considering experimental 56% of water content). Also in this case, a significant correlation between TPP and TEAC/ABTS occurred (y = 0.7292x + 0.4606; *R* ^2^ = 0.920; *p* < .001, at 95% confidence interval; Figure 1b). Finally, as regards the TEAC/DPPH analyses, data ranged 14.7 ± 0.5–78.4 ± 4.1, and 14.2 ± 0.9–121.1 ± 1.2 mmol(TrxEq)/kg DW, for photometric and EPR measurements, respectively, the two methods being in quite good agreement showing a ratio (visible/EPR) by 1.05 ± 0.45.

#### 3.1.3 Pomace samples

Particular attention was paid to pomace material, as a by‐product of olive oil production and as a potential source of antioxidant molecules. The pomaces showed very high antioxidant activities, particularly in samples P15‐D/‐J, that ranged 26.0 ± 1.5–43.7 ± 3.0 g(GAEq)/kg DW (TPP), and 265.4 ± 10.1–388.1 ± 12.0 mmol(TrxEq)/kg DW (TEAC/ABTS; Table 3). In addition to fruits’ quality, the other important factor which strongly affects the content of polyphenols in olive pomace is olive oil production technology. The usage of hot water in three‐phase mill systems brings about a lower antioxidant activity and polyphenol content, as revealed by two samples from the year 2015 that were very dry (P15‐A and P15‐C). This could be reasonably explained suggesting that, the added hot water, works as extragent, moving the polyphenols and other antioxidant species, to waste wasters. Other impact production process can be related to the possible seed removal, which also is a source of antioxidant compounds. Leaving seeds in production process could cause higher antioxidant activity for pomace than for fruits, as seeds were removed from fruits before their analysis.

Finally, it was confirmed an acceptable agreement between TEAC/DPPH analyses, via photometric and EPR measurements, the ratio (visible/EPR) being 1.66 ± 0.39. Moreover, these data confirm that TEAC values measured via the ABTS^•+^ radical cation quenching are usually higher than values measured via the DPPH^•^ radical quenching (Floegel, Kim, Chung, Koo, & Chun, 2011), this being not revealed in olive fruit samples. Furthermore, also in this case, TEAC/ABTS and TPP parameters showed a very good linear correlation in 2014 and 2015 pomace samples *R* ^2^ = 0.853 (y = 8.4483x‐3.9051), improving to *R* ^2^ = 0.948 (y = 8.1276x‐12.7980) when samples P15‐I and P15‐J were excluded (*p* < .001, at 95% confidence interval, in both cases; Figure 1c). These latter samples were from geographical areas different from that of the other samples.

### 3.2 Chromatographic characterization of selected antioxidant components in olive fruits and pomaces

#### 3.2.1 HPLC‐UV: identification and quantification of hydroxytyrosol and oleuropein in olive fruit and pomace samples

The hydroalcoholic (EtOH/H_2_O, 80/20%, v/v) extracts of olive fruits from 2015 harvest showed contents of hydroxytyrosol and oleuropein of the same order of magnitude, ranging 2.4 ± 0.2–6.8 ± 0.3, and 0.7 ± 0.1–9.8 ± 0.4 g/kg DW, respectively (Table 4 and Figure S5). The minimum value for both analytes was found for sample F15‐D (2.4 ± 0.2 g/kg DW). It is interesting to note that sample F15‐J showed a very low content of oleuropein (trace, <0.5 g/kg DW). Found values resulted in great agreement with data previously published for Portuguese varieties (hydroxytyrosol, 1.48–15.76 g/kg DW; oleuropein, 0.34–21.70 g/kg DW; Vinha et al., 2005) and about two/three times higher with respect to Turkish varieties (hydroxytyrosol, 0.04–3.60 g/kg DW; oleuropein, 0.25–3.00 g/kg DW; Arslan & Ozcan, 2011).

**Table 4:** Values of hydroxytyrosol and oleuropein (g/kg DW) in olive fruit and pomace hydroalcoholic (EtOH/H2O, 80/20%, v/v) extracts

Olive fruits	Hydroxytyrosol	Oleuropein	Pomaces	Hydroxytyrosol
F15‐A	3.5 ± 0.2^a^	0.9 ± 0.1^a^	P15‐A	1.2 ± 0.1^a^
F15‐B	4.2 ± 0.1^b^	9.8 ± 0.4^b^	P15‐B	6.4 ± 0.3^b^
F15‐C	6.8 ± 0.3^c^	2.0 ± 0.1^c^	P15‐C	0.4 ± 0.1^c^
F15‐D	2.4 ± 0.2^d^	0.7 ± 0.1^a^	P15‐D	7.7 ± 0.2^d^
F15‐E	4.7 ± 0.2^b^	7.5 ± 0.2^d^	P15‐E	8.0 ± 0.3^d^
F15‐F	4.3 ± 0.3^b^	9.7 ± 0.3^b^	P15‐F	6.1 ± 0.3^b^
F15‐G	4.3 ± 0.1^b^	1.4 ± 0.1^a^	P15‐G	6.1 ± 0.1^b^
F15‐H	3.3 ± 0.2^a^	1.0 ± 0.1^a^	P15‐H	5.5 ± 0.2^e^
F15‐I	6.6 ± 0.3^c^	2.0 ± 0.2^c^	P15‐I	5.4 ± 0.2^e^
F15‐J	4.2 ± 0.2^b^	Trace^e^	P15‐J	5.3 ± 0.2^e^

Note

The values are reported as average ± esd (*n* = 27), and different letters in the same column indicate significant differences (*p* < .05, Tukey's test).

Regarding the olive pomaces from 2015, oleuropein was not quantifiable (trace, <0.5 g/kg DW), whereas hydroxytyrosol ranged 5.3 ± 0.2–8.0 ± 0.3 g/kg DW, excluding the two dry pomace samples (P15‐A and P15‐C), for which the values were much lower (1.2 ± 0.1 and 0.4 ± 0.1 g/kg DW, respectively). The hydroxytyrosol contents correlate quite well with general antioxidant parameters (particularly with TPP; y = 5.1450x + 2.8544; *R* ^2^ = 0.807; *p* < .001, at 95% confidence interval, Figure 1d). Studies indicate that many factors, such as cultivar, geographic origin, pedo‐climatic conditions, agronomical cultivation protocols (i.e., irrigation, fertilization, plant and soil treatments), ripening stage, and postharvest processing, strongly affect the phenolic profile of olive fruits, oil product, and pomace (Uylaşer & Yildiz, 2014). In particular, higher contents of oleuropein, related to the bitter taste to the drupes, have been mainly found in the skin of the fruit, and were reported as related to its ripening stage. Oleuropein undergoes enzymatic processes, by hydrolases and oxidases producing hydroxytyrosol and/or the quinone derivative (Scheme S1). These oxidative reactions also occur during the oil production process (malaxation stage), bringing about oleoside derivatives in olive pomace (Cardoso et al., 2005; Marsilio, 2001; Romero et al., 2004).

#### 3.2.2 HPLC‐MS: identification and quantification of hydroxycinnamic acids and flavonoids in olive fruit and pomace samples

The main components revealed in hydroalcoholic (EtOH/H_2_O, 80/20%, v/v) extracts of olive fruits were chlorogenic acid, rutin, and luteolin, and ranged 3.6 ± 0.5–60.1 ± 2.8, 36.7 ± 4.4–583.9 ± 10.2, and 20.9 ± 1.6–121.0 ± 6.2 mg/kg DW, respectively (Table 5). Quercetin and luteolin‐7‐*O*‐rutinoside were one or two order(s) of magnitude lower with respect to the analogous just mentioned. The concentrations were in agreement with contents reported in previous studies on different varieties: chlorogenic acid, trace – 76.6 mg/kg DW; luteolin, 4.2–269.5 mg/kg DW; rutin, 22.9–242.8 mg/kg DW.

**Table 5:** Values of selected hydroxycinnamic acids and flavonoids (mg/kg DW) for olive fruit and pomace hydroalcoholic (EtOH/H2O, 80/20%, v/v) extracts (trace, <LOQ; nd, not determined < LOD; na, not analyzed)

	Hydroxycinnamic acids				Flavonoids				
	Caffeic	Chlorogenic	Ferulic	*p*‐Coumaric	Rutin	Quercetin	Luteolin	Luteolin−7‐O‐Rutinoside*	Naringenin
Olive fruits									
F14‐A	Trace^a^	18.3 ± 1.9^a^	nd	Trace	41.6 ± 5.2^a^	0.8 ± 0.1^a^	57.6 ± 4.3^a^	na	Trace^a^
F14‐B	Trace^a^	7.4 ± 0.9^b^	nd	Trace	72.6 ± 6.2^b^	2.6 ± 0.2^b^	35.9 ± 3.1^b^	na	Trace^a^
F14‐C	Trace^a^	3.6 ± 0.5^c^	nd	Trace	36.7 ± 4.4^a^	2.7 ± 0.3^b^	55.1 ± 4.6^a^	na	Trace^a^
F15‐A	0.5 ± 0.1^b^	22.6 ± 1.4^a^	nd	Trace	66.6 ± 4.3^a^	1.8 ± 0.1^b^	20.9 ± 1.6^c^	1.6 ± 0.1^a^	Trace^a^
F15‐B	0.5 ± 0.1^b^	60.1 ± 2.8^d^	nd	Trace	410.2 ± 13.4^c^	2.0 ± 0.2^b^	93.6 ± 1.2^d^	8.7 ± 0.1^b^	1.3 ± 0.1^b^
F15‐C	0.6 ± 0.1^b^	25.8 ± 0.9^a^	Trace	Trace	310.1 ± 14.0^d^	2.2 ± 0.3^b^	21.7 ± 2.1^c^	2.6 ± 0.1^c^	Trace^a^
F15‐D	0.7 ± 0.1^b^	18.3 ± 2.1^a^	nd	Trace	178.0 ± 9.1^e^	0.5 ± 0.1^a^	75.0 ± 0.5^e^	3.2 ± 0.3^c^	Trace^a^
F15‐E	1.0 ± 0.1^b^	33.6 ± 0.2^e^	nd	Trace	428.9 ± 2.4^c^	0.9 ± 0.1^a^	86.3 ± 3.7^d^	3.9 ± 0.1^d^	Trace^a^
F15‐F	Trace^a^	29.5 ± 0.1^e^	Trace	Trace	249.3 ± 7.5^f^	0.6 ± 0.1^a^	52.5 ± 0.4^a^	2.3 ± 0.4^c^	Trace^a^
F15‐G	0.9 ± 0.1^b^	23.7 ± 1.5^a^	Trace	Trace	575.6 ± 62.1 ^g^	0.8 ± 0.1^a^	71.1 ± 2.8^e^	6.5 ± 0.9^b^	1.1 ± 0.1^b^
F15‐H	0.6 ± 0.1^b^	39.9 ± 1.3^f^	nd	Trace	583.9 ± 10.2^g^	0.9 ± 0.1^a^	121.0 ± 6.2^d^	7.8 ± 0.7^b^	1.1 ± 0.1^b^
F15‐I	1.0 ± 0.1^b^	19.1 ± 0.1^a^	nd	Trace	374.0 ± 23.7^hr^	1.0 ± 0.1^a^	52.1 ± 11.2^a^	7.2 ± 0.3^b^	1.3 ± 0.1^b^
F15‐J	0.8 ± 0.1^b^	12.2 ± 0.3^g^	nd	Trace	149.9 ± 2.8^e^	1.0 ± 0.1^a^	78.0 ± 0.3^e^	2.3 ± 0.1^c^	1.3 ± 0.1^b^
Pomaces									
P14‐A	451.5 ± 22.3^a^	9.7 ± 1.1^a^	13.3 ± 1.1^a^	33.0 ± 2.9^a^	7.4 ± 1.1^a^	26.2 ± 0.9^a^	57.6 ± 2.1^a^	na	0.9 ± 0.1^a^
P14‐B	523.2 ± 32.1^b^	Trace^b^	34.6 ± 2.1 ^b^	67.1 ± 5.2 ^b^	1.9 ± 0.2^b^	19.7 ± 0.8 ^b^	32.9 ± 1.3^b^	na	0.9 ± 0.1^a^
P14‐C	876.2 ± 45.2^c^	Trace^b^	31.5 ± 1.9 ^b^	31.3 ± 3.1 ^a^	1.3 ± 0.1^b^	33.2 ± 0.5 ^c^	55.1 ± 2.1^a^	na	1.4 ± 0.1^b^
P15‐A	0.7 ± 0.1^d^	Trace^b^	Trace^c^	Trace^c^	4.6 ± 0.5^c^	1.2 ± 0.1^d^	38.1 ± 2.4^b^	Trace^a^	1.7 ± 0.1^b^
P15‐B	53.2 ± 4.6^e^	22.1 ± 1.6^c^	Trace^c^	16.8 ± 1.9 ^d^	9.4 ± 2.6^a^	32.8 ± 3.1 ^c^	230.4 ± 3.0 ^c^	5.0 ± 0.1^b^	1.6 ± 0.1^b^
P15‐C	Trace^f^	Trace^b^	Trace^c^	Trace^c^	5.3 ± 0.2^c^	0.5 ± 0.1^d^	23.6 ± 0.1^d^	Trace^a^	1.0 ± 0.1^a^
P15‐D	214.0 ± 3.9^g^	16.2 ± 0.2^d^	10.0 ± 2.1 ^a^	28.9 ± 1.1 ^a^	10.7 ± 1.0^a^	33.9 ± 1.6 ^c^	246.7 ± 4.8 ^e^	4.6 ± 0.3^b^	1.5 ± 0.2^b^
P15‐E	227.2 ± 18.0^g^	16.8 ± 1.1 ^d^	16.6 ± 1.1 ^d^	34.3 ± 1.5 ^a^	10.6 ± 1.1^a^	36.6 ± 1.9 ^c^	264.9 ± 8.0 ^f^	3.7 ± 0.4^b^	1.8 ± 0.1^b^
P15‐F	35.0 ± 0.1^hr^	20.4 ± 0.5 ^c^	6.1 ± 0.9 ^d^	13.3 ± 0.3 ^e^	161.8 ± 15.2 ^d^	23.0 ± 0.3 ^a^	341.9 ± 3.3 ^g^	6.9 ± 0.7^c^	2.5 ± 0.1^c^
P15‐G	22.5 ± 0.8^i^	39.8 ± 3.5^e^	Trace^c^	7.4 ± 0.4^f^	211.9 ± 12.3^e^	11.9 ± 0.1 ^e^	318.4 ± 3.0 ^hr^	6.1 ± 0.5 ^c^	2.5 ± 0.3^c^
P15‐H	8.6 ± 0.4^j^	47.7 ± 2.6^e^	Trace^c^	8.0 ± 0.8^f^	354.2 ± 24.0 ^f^	9.2 ± 1.2 ^e^	410.9 ± 23.9^i^	8.0 ± 0.9^d^	2.5 ± 0.1^c^
P15‐I	48.9 ± 0.4^k^	25.4 ± 0.5^f^	Trace^c^	18.9 ± 1.2 ^d^	178.9 ± 3.9^d^	21.0 ± 1.7 ^a^	340.0 ± 6.5 ^g^	6.1 ± 0.8^c^	3.3 ± 0.2^d^
P15‐J	37.4 ± 0.5 ^hr^	11.8 ± 0.4^g^	7.2 ± 0.7^e^	13.4 ± 0.6 ^e^	84.2 ± 6.6^g^	25.6 ± 2.1 ^a^	276.9 ± 2.3 ^f^	4.1 ± 0.2^b^	2.2 ± 0.1^c^

Note

The values are reported as average ± esd (*n* = 27), and different letters in the same column (among the same matrix) indicate significant differences (*p* < .05, Tukey's test).

* Luteolin‐7‐*O*‐rutinoside was quantified as luteolin equivalent (mg/kg DW).

The hydroalcoholic (EtOH/H_2_O, 80/20%, v/v) extracts of pomaces confirmed that the contents of hydroxycinnamic acids and flavonoid are related to the production technology. Samples P15‐A and P15‐C (dry pomaces) revealed very low content of all the analyzed species, the highest being the concentration of luteolin (38.1 ± 2.4 and 23.6 ± 0.1 mg/kg DW). Luteolin was the most abundant flavonoid in all the samples of 2015 ranging 230.4 ± 3.0–410.9 ± 23.9 mg/kg DW, two to ten times higher than that of the relevant fruits. The luteolin contents correlate very well with all the antioxidant parameters (TPP: y = 0.1046x + 3.5844; *R* ^2^ = 0.857; TEAC/DPPH(EPR): y = 0.1193x + 3.6854; *R* ^2^ = 0.771; TEAC/DPPH(Vis): y = 5.1450x + 2.8544; *R* ^2^ = 0.807; y = 0.2082x + 3.6645; *R* ^2^ = 0.909; *p* < .001, for all data sets, Figure 1e‐g). A second major component was rutin, ranging 84.2–354.2 mg/kg DW, in samples P15‐F/‐J, relevant to the same milling factory, but not the same primary producer. Looking at the contents of hydroxycinnamic acids, it is possible to identify a synergism between caffeic, chlorogenic, and ferulic acids. Considering that caffeic acid is the precursor of chlorogenic acid (the ester of caffeic acid and quinic acid; Scheme S2a), it appears that chlorogenic acid undergoes enzymatic hydrolysis, bringing about caffeic acid and ferulic acid as metabolites (Scheme S2b). Interestingly, data from 2014 were in good agreement with data from 2015, excluding chlorogenic acid. Studies show that secondary metabolites with high antioxidant capacity play an important role in the protection of plants under oxidative stress (Yan, Cui, Zhao, Chen, & Tang, 2016).

#### 3.2.3 Principal component analysis

**Figure 2:**
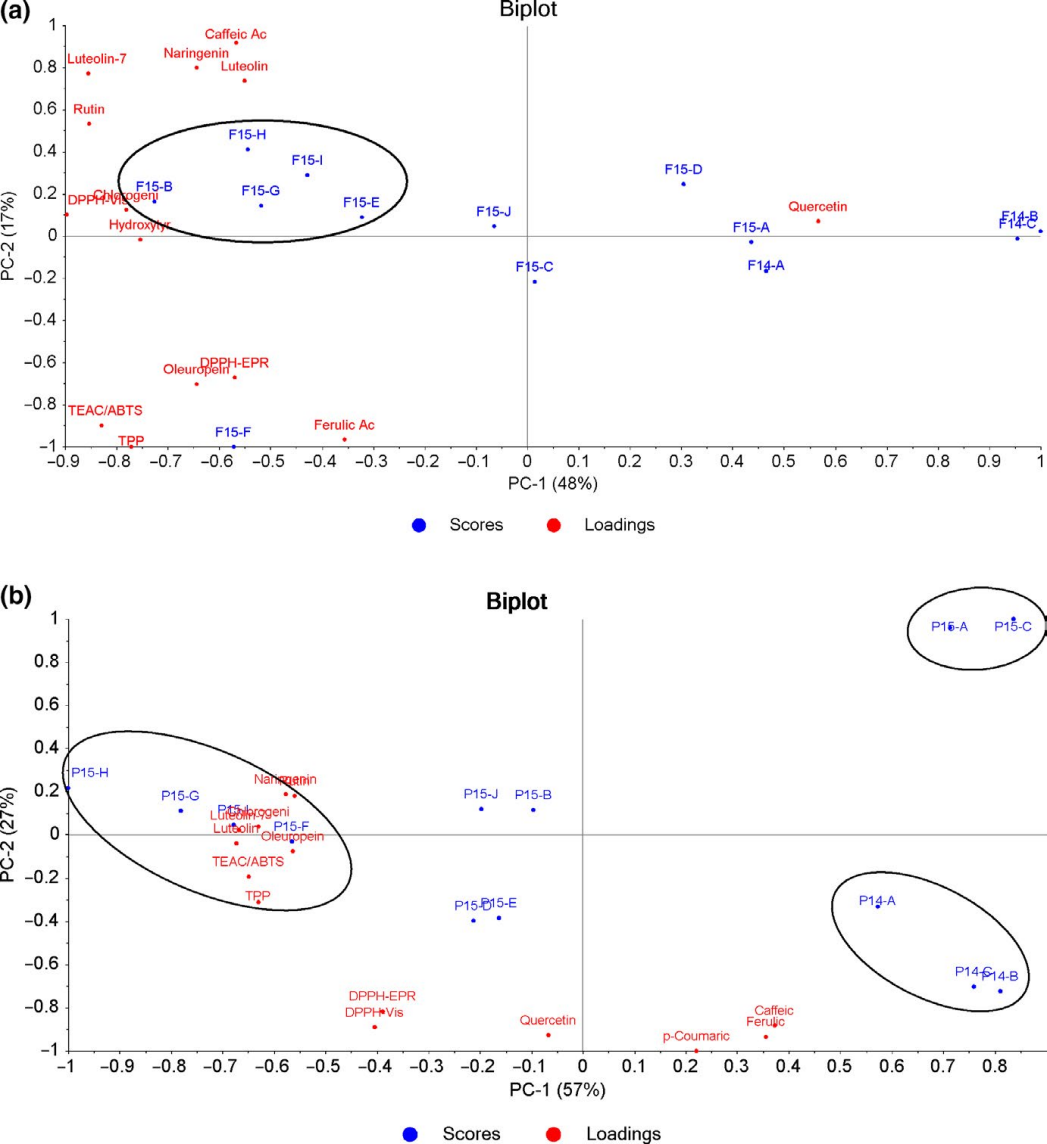
Principal component analysis (PCA) for (a) olive sample and (b) pomace sample data

The data just above commented were confirmed, at a great extent, by the principal component analysis (PCA). The PCA score and loading plots for olive fruits samples were reported in a biplot (Figure 2a) to highlight the correlation between variables and samples. First PC explained the 48% of the total variance. The olive fruits from 2014 harvest appeared separated with higher quercetin contents with respect to the other samples that revealed higher values of TEAC/DPPH, TPP, luteolin, and rutin. The second PC explained 17% of the total variance and separated sample F15‐F, because of its antioxidant parameters values (TPP, TEAC/DPPH, TEAC/ABTS) and oleuropein content.

Pomace samples were also analyzed for the same variables, and the PCA biplot is reported in Figure 2b. It showed that the first and second PCs explained the 57% and 27% of the total variance, respectively. Several groups of samples can be observed revealing a separation with respect to the harvest year and milling technology. The samples 2014 were well separated from samples 2015, on the basis of the higher concentrations of caffeic acid and ferulic acid, both being anticorrelated to the chlorogenic acid. The two samples P15‐A and P15‐C, relevant to dry pomaces from a three‐phase milling process, were also separated because of the low values of TEAC/DPPH. Finally, P15‐F/‐G/‐H/‐I were grouped on the basis of high values of TPP, luteolin, and rutin, and were from the same olive milling factory (using a two‐phase milling process and producing humid pomace). It is noteworthy that olive samples F15‐B and F15‐E were very close to F15‐F/‐G/‐H/‐I from the olive's biplot (Figure 2a), but the first two underwent different treatments at oil phase production and resulted separated in the pomace biplot (Figure 2b): The pressing procedure affected the total antioxidant properties, as well as the chlorogenic acid, luteolin, rutin, and naringenin contents. The multivariate analysis also confirmed the data above commented as regards correlation analysis (TPP vs. TEAC/ABTS, luteolin vs. TPP, luteolin vs. TEAC/ABTS, luteolin vs. TEAC/DPPH).

#### 3.2.4 Cytotoxicity assay: vitality test on NIH3T3 fibroblasts cells

**Figure 3:**
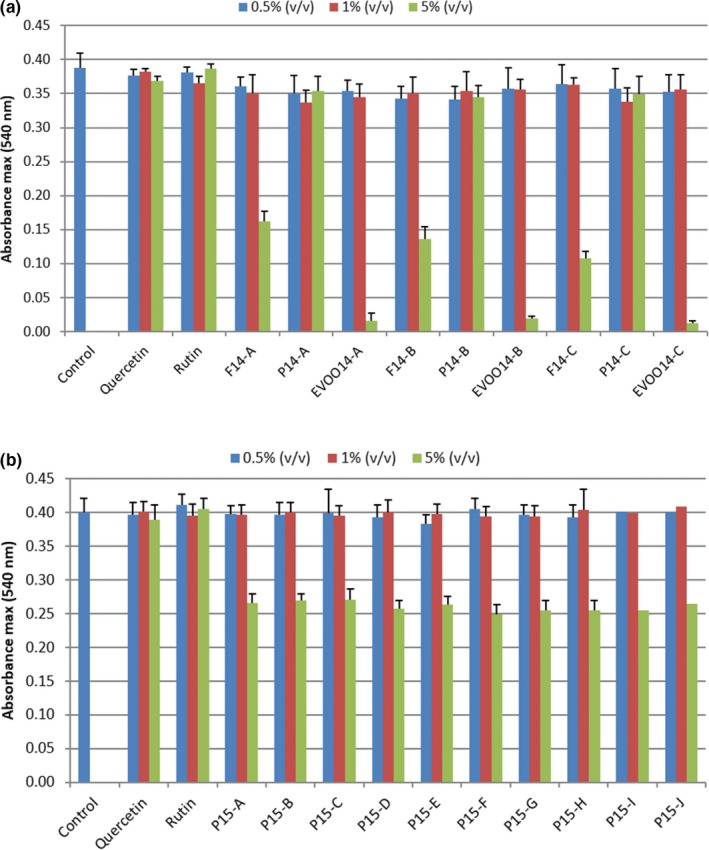
Fibroblast NIH3T3 viability (24 hr) after treatment by hydroalcoholic (EtOH/H2O, 80/20%, v/v) extracts of (a) samples 2014 (0.5, 1.0, and 5.0%, v/v), and rutin and quercetin standard solutions (0.001–0.1 μM), and (b) pomaces 2015 (0.5, 1.0 and 5.0%, v/v). The values are reported as average ± esd (six replicates)

The hydroalcoholic (EtOH/H_2_O, 80/20%, v/v) extracts of olive fruits, EVOOs, and pomaces from the 2014 and 2015 harvests were tested for toxicity on NIH3T3 fibroblasts cells, and compared with pure EtOH and rutin and quercetin standard solutions, at the same extract concentrations (0.001 to 0.1 μM). Quercetin, rutin, and pomace samples from 2014 did not affect NIH3T3 viability, compared to the control at all the tested concentrations (Figure 3a). On the contrary, fruits and EVOOs 2014 revealed toxic effect at 5% (v/v), with a major effect from EVOOs with respect to fruits. For the 2015 harvest samples, none of the tested extracts were toxic at 0.5 and 1% (v/v) concentrations (Figure 3b and Figure S6), whereas 5% (v/v) treated cells showed a great decrease of viability. It was previously reported that quercetin and rutin modified mouse fibroblasts NIH3T3 viability at higher concentrations than those present in the extracts tested in this study (Araújo, de M.B. Costa, Pazini, Valadares, & de Oliveira, 2013; Bonechi et al., 2018). However, this outcome may be reasonably explained as a result of the cumulative and synergic effects of several components and their metabolites.

## 4 CONCLUSIONS

Qualitative and quantitative analyses of olive fruits, olive oils (primary product), and olive pomaces (by‐product from technology) showed multiple factors that influenced the antioxidant properties and polyphenol components. These include genetic factors, fruit maturation stage, agronomical practices, geographical and pedo‐climatic conditions, as well as production technologies (dry and humid pomaces). The results showed that pomace, in particular the humid by‐product, is a promising source of bioactive and antioxidant compounds, without cytotoxic properties. Taking into account the human health benefits of antioxidant polyphenols and considering the importance of olive oil production in the Mediterranean basin, the possibility to utilize olive pomaces as source of nutraceuticals should be a priority. These materials, usually considered as waste products, could be used for the formulation of novel diet supplements and food fortifiers, as well as for applications in cosmetics. This approach allows the valorization of primary and secondary products from *O. europaea* L. and could be considered a model for other agriculture productions (e.g., viticulture, horticulture, cereal crops) to increase the sustainability of agricultural activities.

## CONFLICT OF INTEREST

The authors declare that they have no conflicts of interest.

## ETHICAL STATEMENT

This study does not involve neither human nor animal testing.
