# Supplementary material for: Chemical characterization and antioxidant properties of products and by‐products from Olea europaea L
Source: Food Sci Nutr. 2019 Aug 10;7(9):2907–20. doi: 10.1002/fsn3.1142 (PMC6766567; doi:10.1002/fsn3.1142)
Supplement: Supplementary file 1 [file FSN3-7-2907-s001.docx]

**Chemical characterization and antioxidant properties of products and by-products from *Olea europaea* L.**

Gabriella Tamasi,^1,2^,* Maria Camilla Baratto,^1^ Claudia Bonechi,^1,2^, * Anastasiya Byelyakova,^1^

Alessio Pardini,^1,2^ Alessandro Donati,^1,2^ Gemma Leone,^1,3^ Marco Consumi,^1,3^ Stefania Lamponi,^1,3^

Agnese Magnani,^1,3^ Claudio Rossi^1,2,4^

*^1^Department of Biotechnology, Chemistry and Pharmacy, University of Siena, Via Aldo Moro 2, 53100 Siena, Italy; e-mails:* [gabriella.tamasi@unisi.it](mailto:gabriella.tamasi@unisi.it); [claudia.bonechi@unisi.it](mailto:claudia.bonechi@unisi.it). *^2^Centre for Colloid and Surface Science (CSGI), University of Florence, Via della Lastruccia 3, 50019 Sesto Fiorentino, Firenze, Italy. ^3^National Interuniversity Consortium of Materials Science and Technology (INSTM), Via G. Giusti 9, 50121 Firenze. Italy*

*^4^Operative Unit, University of Siena, Campo Verde, Calabria, Italy*

**SUPPORTING MATERIAL**

**Supplementary** **Table 1.**

Selected details on samples of olive fruits, extravirgin olive oils (EVOOs), and pomaces from the 2014-2015 harvestings, reported in the present study.

| **Sample ID** |  |  | **Sampling Time** | **Milling technology** |
| --- | --- | --- | --- | --- |
| **EVOO 2014** | **Olive Fruit 2014** | **Pomace 2014** |  |  |
| EVOO14-A | F14-A | P14-A | November 2014 | Two-phases * |
| EVOO14-A | F14-B | P14-B | November 2014 | Two-phases |
| EVOO14-A | F14-C | P14-C | November 2014 | Two-phases |
| **EVOO 2015** | **Olive Fruit 2015** | **Pomace 2015** |  |  |
| EVOO15-A | F15-A | P15-A | November 2015 | Three-phases ** |
| EVOO15-B | F15-B | P15-B | November 2015 | Two-phases |
| EVOO15-C | F15-C | P15-C | November 2015 | Three-phases |
| EVOO15-D | F15-D | P15-D | November 2015 | Two-phases |
| EVOO15-E | F15-E | P15-E | November 2015 | Two-phases |
| EVOO15-F | F15-F | P15-F | November 2015 | Two-phases |
| EVOO15-G | F15-G | P15-G | November 2015 | Two-phases |
| EVOO15-H | F15-H | P15-H | November 2015 | Two-phases |
| EVOO15-I | F15-I | P15-I | November 2015 | Two-phases |
| EVOO15-J | F15-J | P15-J | November 2015 | Two-phases |

* Two-phases milling process produces olive oil and humid pomace (that is actually a mixture of pomace and vegetation water, waste-water).

** Three-phases milling process produces olive oil, dry pomace and vegetation water (waste-water).

^§^ Samples collected at the same oil milling plant (different from §§)

^§§^ Samples collected at the same oil milling plant (different from §).

**Supplementary** **Figure 1.** Examples of calibration curves for (a) Folin-Ciocalteu TPP assay, (b) TEAC/ABTS assay, (c) TEAC/DPPH assay via photometric measurements, and (d) TEAC/DPPH assay via EPR measurements.

(a)

(b)

(c)

(d)

**Supplementary** **Figure 2.** (a) Overlap of chromatograms recorded from the injection of standard solutions for building calibration curves (resveratrol was used as internal standard). Example of (b) hydroxytyrosol and (c) oleuropein calibration curves, via HPLC-UV.

(a)

(b)(c)

**Supplementary** **Figure 3**. Examples of HPLC-MS chromatogram for determining hydroxycinnamic acids and flavonoids in a standard solution (1 mg/L, each species), and relevant calibration curves.

(b) (c)

(d) (e)

(f) (g)

(h) (i)

**Supplementary** **Figure 4.** Average monthly (a) temperature (°C), and (b) humidity (%) for years 2013, 2014 and 2015 (Source: Meteo archive, http://www.ilmeteo.it/portale/archivio-meteo/).

(a)

(b)

**Supplementary** **Figure 5**. HPLC-UV chromatograms of (a) olive fruit samples F15-F and F15-J (overlay, in the range 5-25 min, retention time), and (b) pomace sample P15-D.

(a)

(b)

**Supplementary** **Figure 6**. Fibroblast NIH3T3 viability (24 h) after treatment by hydroalcoholic (EtOH/H_2_O, 80/20%, v/v) extracts of (a) EVOOs 2015 (0.5, 1.0 and 5.0%, v/v), and (b) fruits 2015 (0.5, 1.0 and 5.0%, v/v). The values are reported as average ± esd (six replicates).

(a)

(b)

**Supplementary** **Scheme 1**. Scheme of enzymatic processes occurring in olive ripening process.

**Supplementary** **Scheme 2**. Schematic reaction for the (a) hydrolysis of chlorogenic acid that brings about caffeic acid and quinic acid; and (b) formation of ferulic acid from *trans*-cinnamic acid.

(a)

(b)
